# Hepatocyte paraffin 1 and arginase-1 are effective panel of markers in HBV-related HCC diagnosis in fine-needle aspiration specimens

**DOI:** 10.1186/s13104-020-05230-y

**Published:** 2020-08-20

**Authors:** Bita Moudi, Hamidreza Mahmoudzadeh-Sagheb, Zahra Heidari

**Affiliations:** 1 Infectious Diseases and Tropical Medicine Research Center, Resistant Tuberculosis Institute, Zahedan University of Medical Sciences, Zahedan, Iran; 2 Department of Histology, School of Medicine, Zahedan University of Medical Sciences, Zahedan, Iran

**Keywords:** Arg-1, HepPar-1, Hepatocellular carcinoma, Immunohistochemistry

## Abstract

**Objective:**

In order to make successful treatment for HBV-related hepatocellular carcinoma, an early diagnosis is necessary. In this research we aimed to evaluate the IHC staining pattern of Hepatocyte paraffin 1 and arginase-1 and their performance in early diagnosis of HCC. The incidence of HepPar-1 and Arg-1 were evaluated by IHC in 121 patients (HBV, HCC, HBV + HCC) and 30 healthy subjects.

**Results:**

Arg-1 had significantly increased sensitivity in identification of HBV + HCC patients compared to HepPar-1 (P < 0.001). The sensitivity of arginase-1 is 96.3% whereas, the sensitivity of HepPar-1 is 72.7%. Arg-1 had higher specificity in identification of HBV + HCC patients compared to HepPar-1 (P < 0.05). With one positive marker, the sensitivity, the specificity and the positive predictive values and negative predictive value were 84.3%, 82.4%, 88.6% and 85.4% respectively. Also with one positive marker, the sensitivity and negative predictive value were significantly higher compared to the both 2 positive combinations. It was concluded that Arg-1 can improves the ability to detect HBV + HCC patients when compared with HepPar-1. When, both markers being positive, the specificity and PPVs of this combination were fairly higher. Concurrent use of these two proteins may be one of the best HCC detection patterns in needle specimens.

## Introduction

Many HCC patients die because of their late diagnosis. Therefore, rapid identification of the disease is an important factor in improving the survival rate of the patient [1]. Identification of cancer-specific biomarkers, based on antibody-antigen interactions (immunohistochemistry, IHC), is helpful in the diagnosis of cancer pathology [2]. There is no definitive specific biomarker for HCC in the early stages, so far and it seems that focusing on multiple antibodies simultaneously using IHC can be helpful. These biomarkers must function differently under various pathological conditions to identify the types of HCC cases (HCV and/or HBV related cancer) [3–5].

Recently, monoclonal antibodies have been designed that can bind exclusively to hepatocyte epitopes. One of these antibodies is Hepatocyte paraffin 1 (HepPar-1). Expression of the HepPar-1 in the liver tissue is significantly correlated to the level of cancer progression [6]. HepPar-1 has been reported as most sensitive and specific immunohistochemical marker for HCC [7]. So far, in only one study, the sensitivity of this antibody has not been convincing (50% or less). Kakar et al. faced significant number of false-negative diagnoses as they began to identify cancer patients with poorly differentiated HCCs [8]. HepPar-1 along with some other biomarkers can also be helpful in the diagnosis of nonhepatocellular tumors [9, 10].

It seems that HepPar-1 antigen is one of the most important factors in the arginine and urea metabolism cycle [11]. Another enzyme involved in this pathway along with HepPar-1, is arginase-1 (Arg-1). This enzyme is produced in normal liver tissue and has a remarkable specificity in the diagnosis of liver lesions [12]. Arg-1 mainly concentrates in periportal hepatocytes [13]. Chrzanowska et al. found that patients with cirrhotic nodules and HCCs had lower levels of Arg-1 compared to the healthy subjects.

The aim of this study was to investigate the immunohistochemical expression pattern of HepPar-1 and Arg-1 in patients with HBV, HCC and HBV + HCC. The question was that, whether these two markers could be helpful in early detection of liver cancer in HBV infected patients?

## Main text

### Material and methods

In this study, 40 patients with HBV, 41 patients with liver cancer (early HCC), 40 patients with both hepatitis and early HCC were enrolled. They formed patient groups and diagnosis of HBV and/or HCC in all people were done according to World Health Organization criteria [14]. The tumors were single and smaller than 5 cm in size. Vascular invasion was not observed in any of the patients. Patients with hepatitis B infection were HBsAg^+^ and HBV-DNA^+^. Thirty healthy individuals (C), who were intended to donate liver, were selected as the control group. Their serological markers were negative for hepatitis B, C infections and cancer. Serum concentrations of ALT were also normal in these subjects. Sampling was performed at two main liver disease treatment centers (Shiraz and Tehran, Sep2015-May2016). The demographic data were reported in Table 1. All subjects in this study provided written and informed consent.

**Table 1:** Demographic and clinical data of control (C), HBV infected (HBV), hepatocellular carcinoma (HCC) and HBV-related HCC (HBV + HCC) groups

Parameters	C, N(%)	HBV, N(%)	HCC, N(%)	HBV + HCC, N(%)	P
Age (years)					P = 0.161 F = 1.740
Mean age	33 ± 6.216	53.85 ± 9.582	55.44 ± 10.305	57.13 ± 9.819	
Age range	37–61	31–71	30–72	37–72	
	Median	Median	Median	Median	
	51.50 years	58 years	56 years	59 years	
Sex					P = 0.738 F = 0.413
Male	24(80.0)	28(70.0)	32(78.0)	29(72.5)	
Female	6(20.0)	12(30.0)	9(22.0)	11(27.5)	
Hepatocellular carcinoma					
Well or moderately differentiated			37(90.2)	35(87.5)	
Poorly differentiated	-	-	4(9.8)	5(12.5)	
HCC grading					
Early			39(95.1)	38(95.0)	
G1	–		1(2.4)	2(5.0)	
G2–G3		–	1(2.4)	0	
Total bilirubin (μ mol/l)	15.43 ± 5.65	18.76 ± 6.75	28.45 ± 12.24	33.10 ± 11.77	
ALT (U/L)	26.23 ± 10.90	45.76 ± 32.03	88.25 ± 95.32	117.76 ± 102.54	
AFP (ng/mL)	2.12 ± 1.14	3.12 ± 2.79	421.21 ± 104.33	534.54 ± 420.76	
Serum HBV DNA level					
Mean, log IU/mL (1SD)	–	7.6 ± 0.8	–	7.8 ± 0.1	
HBs-Ag positive	–	40(100.0)	–	40(100.0)	
HBe-Ab positive	–	12(30.0)	–	15(37.5)	

Liver tissue samples were fixed and stored in formalin buffered solution. A paraffin block was prepared from each sample. Routine histological and pathological tests and immunohistochemical evaluation were then performed. The immunohistochemical technique was performed using primary antibodies to HepPar-1 (Thermo Scientific), Arg-1 (SANTA CRUZ, USA) and secondary antibodies (SANTA CRUZ, USA) according to the instructions of manufacturer. The immunohistochemical technique was performed as follows: briefly, at first, sections were deparaffinized with xylene. The sections were then rehydrated to facilitate staining. Inhibition of endogenous peroxidases and removal of antigens were performed by H2O2 solution (0.3%) and treatment by sodium citrate buffer along with autoclave (Prestige Medical, Model Number 210003, Iran), respectively. In this stage, sections were treated by primary antibodies and secondary antibodies, respectively.

Staining density and intensity of sections were sorted by following pattern: sections without stained cells, or less than five percent, sections with mild amounts of stained cells (< 5–25%), sections with temperate amounts of stained cells (25–75%), sections with vigorous amounts of stained cells (> 75%) for density. Intensity was classified into 4 classes: 0 as sections with absent the staining, 1 as sections with weak staining, 2 as sections with moderate staining, 3 as sections with strong staining [15].

#### Statistical analysis

Statistical analysis was done using SPSS program version 20 statistical software package. To compare the statistical data nonparametric Mann–Whitney test, Kruskal Wallis, One-way ANOVA, Chi-square and Fisher’s exact tests were used. P values less than 0.05 were considered as statistically significant. All P values were two-sided.

### Results

Clinical and demographic data of all subjects were summarized in Table 1. The groups were matched for age and gender and there was no statistically significant difference in these parameters (P > 0.05). Pattern of staining and clinical features of groups were not significantly correlated.

Expressions of the HepPar-1 and Arg-1 biomarkers were compared between the four groups (Table 2). HepPar-1 and Arg-1 are immunohistochemically expressed mainly in the cytoplasm of hepatocytes. According to Table 2, HBV patients with HCC had decreased levels of Arg-1 compared with the patients with only HBV infection and healthy subjects (P < 0.001) and also patients with only HCC (P = 0.017). Moreover, HBV patients with HCC had lower levels of HepPar-1 than patients with only HBV infection (P = 0.015) and healthy subjects (P < 0.001). As mentioned in Table 2, no statistically significant relationship was observed between HBV + HCC and only HCC in regard to the expression levels of HepPar-1 (P = 0.118).

**Table 2:** Comparing the expression levels of Arg-1 and HepPar-1 in liver tissue samples of HBV-related HCC, HBV infected, HCC and healthy control groups

Group	N	Arg-1 Positive, (Mean ± SEM)	P value	HepPar-1 Positive, (Mean ± SEM)	P value
C	30	13.02 ± 1.87	P < 0.001	9.70 ± 2.54	P < 0.001
HBV	40	9.26 ± 1.84		5.47 ± 1.03	
HCC	41	7.65 ± 1.59		5.17 ± 1.20	
HBV + HCC	40	6.26 ± 2.06*^,^**		4.30 ± 1.06^¥,#^	

*P < 0.001, Compared with C, HBV groups. Bonferroni correction P_BC_ < 0.001

**P = 0.017, Compared with HCC groups. Bonferroni correction P_BC_ = 0.02

^¥^P = 0.015, Compared with HBV groups. Bonferroni correction P_BC_ = 0.018

^#^P < 0.001, Compared with C groups. Bonferroni correction P_BC_ < 0.001

Sections with normal liver tissue showed a diffuse and strong pattern of staining of both biomarkers (Table 2). HBV patients with HCC had decreased number of HepPar-1 and Arg-1 positive cells compared with the patients with only HBV infection, only HCC and healthy subjects (P < 0.001).

Additional file 1: Figure S1A–D and Additional file 2: Figure S2A–D showed the immunohistochemical staining of HepPar-1 and Arg-1 positive cells in patients and healthy subjects. As expected, HBV patients with HCC had lower levels of HepPar-1 and Arg-1 expressions than patients with only HBV infection and only HCC (P < 0.001).

The sensitivity of Arginase-1 for diagnosis of cancer in HBV infected patients was more than that of HepPar-1 (P < 0.001). The sensitivity of arginase-1 was 96.3% whereas of HepPar-1 was 72.7%. This was also seen in the diagnostic specificity of Arg-1 compared with the HepPar-1 (P < 0.05). In regard to the diagnosis of cancer in people with hepatitis B infection, Arg-1 showed increased positive predictive value (PPV) amount compared with HepPar-1. Also, Arg-1 showed more reliable negative predictive value (NPV) in diagnosis of HCC in HBV patients than that of HepPar-1.

Overall, the combinations of the 2 positive markers for HCC detection were listed in Table 3. When at least 1 marker was positive, regardless of which one, the sensitivity and NPV were significantly higher compared to the both 2 positive combinations. When, both markers being positive, the specificity and PPV values were fairly higher.

**Table 3:** Diagnostic accuracy for detection of hepatocellular carcinoma using one or two markers

	Sensitivity (%)	Specificity (%)	PPV (%)	NPV (%)	Accuracy
One marker					
Arg-1	96.3	88.4	95.5	87.5	88.4
HepPar-1	72.7	84.3	82.2	64.3	77.8
Two markers					
All 2 positive	70.6	100	100	77.6	89.4
At least 1 positive	84.3	82.4	88.6	85.4	72.2

*Arg-1* arginase-1, *HepPar-1* hepatocyte paraffin 1, *NPV* negative predictive value, *PPV* positive predictive value

### Discussion

Histological changes caused by HCC are varied in different patients that cause enormous problems in diagnosis. Therefore, accurate detection methods are necessary to assuredly diagnosis of HCC in an early stage.

HepPar-1 is one of the key factors in the urea metabolism cycle and can be highly sensitive and specific in detect of hepatocytes [16]. Arg-1 is mainly produced in the liver tissue, the main objective of this study was to evaluate the expression pattern of Arg-1 and HepPar-1 in patients with liver cancer [17].

The staining pattern of hepatocytes by Arg-1 was mainly diffuse cytoplasmic and patchy nuclear reactivity, both in normal and HCC liver samples. Given that the role and function of Arg-1 in the nucleus are unknown and its expression is poor, in many studies scientists neglect it and only consider the expression of Arg-1 in the cytoplasm [18].

In the current study, HBV infected patients with HCC had significant lower levels of HepPar-1 and Arg-1 than patients with only HBV infection. These proteins can partially determine the likelihood of cancer in people with hepatitis B infection. The expression of HepPar-1 and Arg-1 is associated to the risk of HBV-related HCC, as both of them have been reduced in patients with HBV + HCC. Also, Arg-1 was more specific and sensitive than HepPar-1 and could be a more suitable biomarker for increase the specificity and sensitivity to an acceptable level.

As revealed by the findings of this study, in all groups, Arg-1 has been shown to be more sensitive to detection of cancer than HepPar-1. The results in the field of HepPar-1 function in cancer diagnosis are almost similar to those of other studies, Fan et al. [19], Fu et al. [20], Wang et al. [10] and Benjamin et al. [18]. On the other hand, all cancer patients showed a diagnostic response to both proteins and apparently, Arg-1 can easily and reliably replace HepPar-1 in cancer diagnostic processes. Studies by Yan et al. [18] and Dana et al. [21] also confirm this hypothesis.

The Arg-1 specificity in the diagnosis of cancer was somewhat higher than that of HepPar-1. So, diagnosis of HCC by HepPar-1, must be handled with care. Given that, distinguishing of HBV-related HCC in early stages is crucial, therefore in order to confirm the diagnostic specificity of Arg-1 rigorously, the expression level of both proteins was evaluated especially in the groups that were potential prone to HCC, including HBV infected patients. Of note, healthy controls often express HepPar-1 in a way that Fasano et al. have found that it was produced in subjects without HBV or HCC [22].

Our findings in current study indicate the important diagnostic specificity of Arg-1 in cancer patients on fine-needle aspiration specimens. Also, Arg-1 evaluation showed that this protein could be highly sensitive in identifying hepatocytes [18]. Furthermore, proper combination of HepPar-1 and Arg-1 improved the accuracy of cancer detection in people with hepatitis B which can helpful in disease control. HBV patients with cancer were diagnosed with a 100% specificity, when using a combined model of Arg-1 + HepPar-1. One of the complex issues in the detection of liver malignancies is diagnosing hepatic failures which are susceptible to advanced stages of liver disease. In this study, the Arg-1 antigen partially resolved this problem using an immunohistochemical panel because it was able to be expressed as a specific marker of hepatocyte cells in all groups with a clear pattern.

Another point that was highlighted by the findings of this study was that the combined model included Arg-1 + HepPar-1, improved the process of cancer diagnosis of HBV-related HCC, especially in fine-needle aspiration samples with too small cell count to evaluate the status of the sample and disease.

### Conclusion

This study emphasized that Arg-1′s efficacy in HCC diagnosis in patients with HBV infection is more accurate than HepPar-1. Arg-1 was better able to detect hepatocyte cells with a higher sensitivity and specificity which had led to improved cancer detection in hepatitis B infected patients. The identification of Arg-1 as an immunohistochemical marker of HCC may lead to its development as a useful diagnostic tool in routine surgical pathology practice. Finally, the proper combination of HepPar-1 and Arg-1 is more useful for the management of the liver malignancy in different stages.

## Limitation

The current study has several limitations. Despite the results obtained in this research, until larger studies of HepPar-1 and Arg-1 are evaluated on fine-needle aspiration samples, the probability of any changes in antibody-antigen reactions related to the HepPar-1 and Arg-1 induced by some situations in histological preparations, must be studied at least. Another ambiguity about Arg-1 that needs to be addressed is that this antigen is mainly detectable in the cytoplasm using IHC and rarely in the nucleus [18]. In the current study and studies by Yan et al. [18] and Dana et al. [21], the cytoplasmic staining pattern of this protein has been considered only. However, in some normal and/or HCC liver tissue, Arg-1 is expressed both in the nucleus and in the cytoplasm but, the significance of Arg-1 expression in the cell nucleus is still unknown.

## Supplementary information

**Additional file 1: Figure S1.** Arginase-1 expression in control (A), HBV (B), HCC (C) and HBV + HCC (D) liver tissue (Immunperoxidase ×400). Arginase-1 positive expression in hepatocytes (white arrowheads) are shown.

**Additional file 2: Figure S2.** Hepatocyte paraffin 1 expression in control (A), HBV (B), HCC (C) and HBV + HCC (D) liver tissue (Immunperoxidase ×400). Hepatocyte paraffin 1 positive expression in hepatocytes (white arrowheads) are shown.

## Data Availability

All data generated or analyzed during this study are included in this published article and are available from the corresponding author.

## Abbreviations

HBV: *Hepatitis B* virus infection
HCC: Hepatocellular carcinoma
Hep-Par 1: Hepatocyte paraffin 1
Arg-1: Arginase-1
