# Supplementary figures and images for: Hepatocyte paraffin 1 and arginase-1 are effective panel of markers in HBV-related HCC diagnosis in fine-needle aspiration specimens
Source: BMC Res Notes. 2020 Aug 20;13:388. doi: 10.1186/s13104-020-05230-y (PMC7450594; doi:10.1186/s13104-020-05230-y)

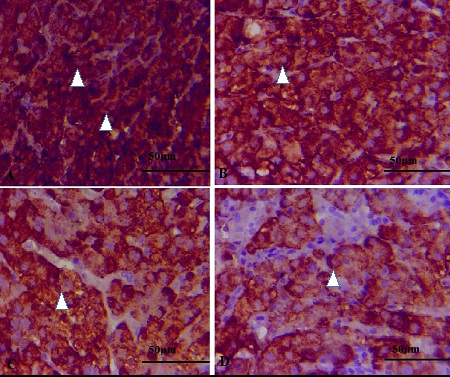

Supplement: Supplementary file 1 — Additional file 1: Figure S1. Arginase-1 expression in control (A), HBV (B), HCC (C) and HBV + HCC (D) liver tissue (Immunperoxidase ×400). Arginase-1 positive expression in hepatocytes (white arrowheads) are shown. [file 13104_2020_5230_MOESM1_ESM.jpg]

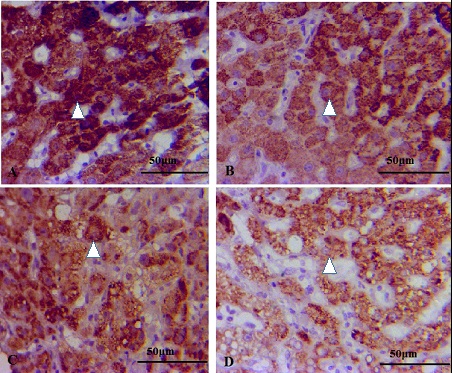

Supplement: Supplementary file 2 — Additional file 2: Figure S2. Hepatocyte paraffin 1 expression in control (A), HBV (B), HCC (C) and HBV + HCC (D) liver tissue (Immunperoxidase ×400). Hepatocyte paraffin 1 positive expression in hepatocytes (white arrowheads) are shown. [file 13104_2020_5230_MOESM2_ESM.jpg]
